# The Use of Nanobiotechnology in Immunology and Vaccination

**DOI:** 10.3390/vaccines9020074

**Published:** 2021-01-21

**Authors:** Reza Keikha, Karim Daliri, Ali Jebali

**Affiliations:** 1 Infectious Diseases and Tropical Medicine Research Center, Resistant Tuberculosis Institute, Zahedan University of Medical Sciences, Zahedan 9816743463, Iran; rezakeikha.md@gmail.com; 2 Department of Pathology, Faculty of Medicine, Zahedan University of Medical Sciences, Zahedan 9816743463, Iran; 3 Department of Genetics, Shiraz University of Medical Sciences, Shiraz 1433671348, Iran; karimdaliri@gmail.com; 4 Molecular Medicine Research Center, Research Institute of Basic Medical Sciences, Rafsanjan University of Medical Sciences, Rafsanjan 7717933777, Iran

**Keywords:** nanotechnology, nanostructures, immunology, vaccination

## Abstract

Nanotechnology uses the unique properties of nanostructures with a size of 1 to 200 nanometers. Different nanoparticles have shown great promise for the production of new vaccines and drugs. Nanostructures can be used to deliver immunological compounds more effectively than microstructures to target sites. Different nanostructures can be applied to form a new generation of vaccines, adjuvants, and immune system drugs. The goal of nanotechnology is to better respond to a wide range of infectious and non-infectious diseases.

## 1. Introduction

Biomolecules such as oligonucleotides, polysaccharides, and proteins are antigenic and all are nanometer-sized [1]. Table 1 shows the size distribution of some immunological structures [2]. In this review article, we will discuss which types of nanoparticles are good for vaccination. Their size, shape, charge, porosity, and hydrophobic properties are important. Interestingly, there is a relationship between nanoparticle size and bioactivity [3] (Table 2). We look at the role of nanotechnology in the development of vaccines and immunosuppressants. It should be noted that the field of nanotechnology is very broad and we have tried to show only immunological applications of nanomaterials. Inactive vaccines usually use adjuvants that increase the quantity and quality of cellular and humoral immune responses. Although nanoparticles enhance the delivery of antigens to the immune system, they also boost immune responses [4]. Here, we introduce some nanoparticles such as virus-like particles (VLP) and squalene-based oil-in-water emulsions such as MF59, which have been used for decades, but other nanoparticles are still in the early stages of development (Table 3).

## 2. VLP-Based Vaccines

VLPs are very diverse nanoparticles that range in size from 20 to 150 nanometers. There are several benefits to using VLPs, including unique nanometer size, symmetrical shape, uniformity, and stable structure [5]. Several applications for VLPs have been reported such as vaccination against infectious diseases and even cancer. Recently, protection against chronic inflammation diseases, such as high blood pressure, Alzheimer’s disease, and rheumatoid arthritis by VLPs have been reported. Interestingly, VLPs have also been used in the preparation of vaccines against drug addiction [6].

In terms of vaccination, VLPs are divided into two classes: (a) VLPs that are subunits of viral surface proteins [7]; (b) artificial VLPs produced by chemical synthesis [8,9]. Furthermore, VLPs can be distinguished from viruses by their lack of genetic material and their inability to reproduce or perform genetic recombination. More than 20 to 30 different VLPs are currently at the clinical or pre-clinical stage [10]. Although VLPs can be prepared without modification or genetic engineering, they can be conjugated with antigenic peptides or ligands [11].

Toll-like receptor (TLR) ligands, cell receptors, or other biologically active mediators can also be linked to VLPs to enhance their efficacy. In a human clinical trial, a VLP was loaded with synthetic A-type CpG-oligonucleotides (CpGODNs) and peptide epitopes derived from melanoma-associated antigens, such as melanoma antigen recognized by T cells 1 (MART1). It has been shown that this VLP can activate multifunctional central memory T cells and cytotoxic T lymphocytes (CTLs) and secretes tumor necrosis factor-α (TNF), interferon-γ (IFNγ), and interleukin 2 (IL-2). These viral nanoparticles have been uptaken more by antigen-processing cells (APCs) [12].

**Figure 1:**
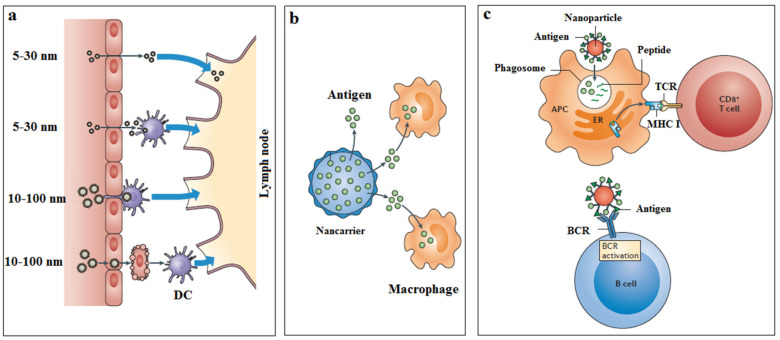
The delivery of nanoparticles across biological barriers and access to the lymphatics in different ways (**a**). The release of vaccine antigen to macrophage; (**b**) a depot effect, which promotes the persistence, the stability, the conformational integrity, and the gradual release of vaccine antigens. The activation of B cells and T cells by nanoparticles (**c**) by TLR, MHC class I, TCR, and BCR. APC, antigen-processing cell; DC, dendritic cell; TLR, Toll-like receptor; TCR, T cell receptor; BCR, B cell receptor.

It is noteworthy that small nonenveloped VLPs (size 25 to 40 nanometers) overcome better tissue barriers and reach the lymph nodes. Larger VLPs (>100 nanometers) typically accumulate at the injection site and hardly reach the lymph nodes. Nanoparticles also appear to be more suitable for targeting cells than microparticles [13]. For example, when nanoparticles are coated with CD209 monoclonal antibodies, they can easily bind to dendritic cells (DC) better than microparticles. While most extracellular proteins are processed through the MHC class II pathway, the VLP nanoparticles are processed in both MHC class I and II, leading to the proliferation of both CD4^+^ T cells and CD8^+^ T cells [14,15] (Figure 1).

Two VLP-based vaccines are currently licensed. One prevents the hepatitis B virus (HBV) and decreases the risk of hepatic carcinoma [16,17], and the other prevents the human papillomavirus from causing cervical cancer [18]. The HBV vaccine with alum adjuvants has a major limitation. Alum mainly activates T-helper 2 (TH2) and it cannot activate TH1 [19]. In a recently developed HPV vaccine, monophosphoryl A (MPL) lipid A added to induce TH1 and CTLs [20]. Although current VLP-based vaccines are effective, they also have some limitations [21]. There is insufficient evidence to support the safety of vaccination programs, which calls for additional studies. VLPs and other nanoparticle-based technologies offer novel solutions for the powerful and long-term induction of humoral and cellular immune responses. They are also useful for protection and prevention against chronic infections and highly mutable infections such as influenza and coronavirus [6,22,23].

## 3. Artificial VLP

Developing artificial VLPs using lipopeptide monomers to strengthen and stabilize the three-dimensional structure of protein antigens is a novel strategy. An artificial VLP does not require recombinant DNA technology or the expression and purification of monomer proteins. One example of this platform is an artificial VLP for gp120 HIV. It displays a peptide-mimetic epitope derived from the V3-variable loop of gp120 HIV [24]. This VLP can be connected to different MHC class II molecules and activates TLR2. Interestingly, the immunization of New Zealand white rabbits with these artificial VLPs has led to the production of neutralizing antibodies [25,26].

## 4. Nanoparticle-Based Vaccine Carrier

Several types of nanoparticles have been applied as vaccine carriers to trap specific antigens. Examples of nanoparticles used as carriers in vaccination are poly (lactide-glycolide) nanoparticles (PLGA) [27], hydrogel polymers or “nanogels” [28], cationic liposomes [29], and cholesterol-bearing hydrophobized pullulan (CHP) [30]. In nanotechnology, polymers have also been utilized as nanospheres, nanoparticles, and nanocapsules [31]. PLGA vaccine carriers are widely tested in animal models and clinical applications as a matrix to be able to release drugs gradually [32]. In vaccine production, pegylated PLGA (150 to 200 nm) is used to encapsulate the hepatitis B surface antigen (HBsAg) [33]. For example, a heat-degradable hydrogel consisting of pegylated PLGA (less than 100 nm) was injected subcutaneously in mice to release HBsAg at the injection site. Biodegradable polymer nanoparticles have also been shown to be safe and biocompatible for application in vaccine technology [34,35,36].

## 5. Self Assemble Peptide Nanoparticles (SAPNs)

SPANs are created by the algorithmization of structural proteins to create a platform with repeating units [37]. The novel structure, expressed in *Escherichia coli*, has a two-part polypeptide chain that eventually forms a nanometer structure [38]. Nearly 180 peptide units are placed together as a nanoparticle and can stimulate the immune system. The scaffold shows antigens and activates immune cells, leading to a significant increase in the production of antibodies [39]. SAPNs can be used to produce a wide range of different antigens including aqueous peptide annular epitopes, and can successfully produce antibodies. This structure has been used to prepare vaccines for the acute respiratory syndrome coronavirus (SARS-CoV) and avian influenza virus [38,40].

## 6. Cationic Liposomes

Cationic liposomes (typically with a particle diameter of 200 to 1000 nm depending on the formulation) are used to encapsulate the antigens. They absorb antigens and release them into immune cells for a long time. The positive charge and lipid composition improve their efficiency. The used cationic lipids include quaternary ammonium compounds (dimethyl dioctadecyl ammonium bromide (DDA)), 1,2-dioleoyl-3-trimethyl-ammonium propane (DOTAP), cholesterol derivatives (dimethylaminoethanhanecarbamoyl-cholesterol), imidazolium compounds 1-[2-(oleoyloxy) ethyl]-2-oleyl-3-(2-hydroxyethyl) imidazolinium chloride (DOTIM)), and diC14 -amidine-based compounds, as well as other immunostimulants such as trehalose dibehenate (TDB) which is a synthetic analog of trehalose dimycolate [29,41].

## 7. Nano-Emulsion

Nano-emulsions are a mixture of water in oil and are composed of solvents and surfactants. An example of a nano-emulsion is MF59 which is composed of squalene oil in combination with polymorphic 80 (Tween 80) and sorbitan triolate (Span 85) [42]. In Europe, the MF59 vaccine is licensed for use in influenza vaccines and can be intramuscularly administered [43]. Its functional mechanisms appear to include increased antigen uptake, the release of inflammatory cytokines, accumulation of monocytes, and granulocytes at the injection site. MF59 is better than alum because it provides both humoral and cellular immune responses. Increased reactivity and pain at the injection site have been described after MF59 administration. This has been attributed to increased inflammation due to enhanced immune response [44,45,46,47].

W805EC nano-emulsion is composed of soybean oil and has been intramuscularly tested in animals and humans, resulting in a strong mucosal, humoral, and cellular immune response. The unique activity of this type of nano-emulsion is to maintain the structure and their positive charge, facilitating the connection to the mucosal membrane. Their size and the positive potential coefficient of nano-emulsion lead to penetrating the mucosal layer and binding to the cell membrane. Due to the high efficiency of nano molecules, they can be used to prepare vaccines for a variety of viruses, bacteria, and fungi. Furthermore, nano-emulsions have no toxicity according to extensive human and animal experiments [48,49].

## 8. Other Nanoparticles

In addition to the above nanostructures, researchers are testing other intelligent nanostructures for use in vaccines and immunology. For example, calcium phosphate or hydroxyapatite nanoparticles can be a good platform for vaccination [50]. Specialized nano-platforms can also be made using plasmids and DNA technology [51,52]. A new strategy in vaccine design is to target B-cells by derivatizing the nanoparticle carrier with TLR ligands [53]. The surface manipulation [54,55] and the use of detector molecules such as aptamers [56] are examples of targeting. Of course, each method has its advantages and limitations [57]. Another technology is bacterial outer membrane vesicles (OMVs)-based vaccines [58], presenting the antigenic stable chimeric fusion protein of the H1-type haemagglutinin (HA) of influenza A virus and the receptor-binding domain (RBD) of MERS-CoV (OMVs-H1/RBD). The OMVs-based vaccines presenting viral antigens provide a safe and reliable approach to protect against two different viral infections [59].

In addition to the stimulation of the immune system, nanotechnology can be used therapeutically to inhibit harmful immune responses that occur in allergies, autoimmunity, and transplant rejection [60]. Table 4 shows the immunosuppressive effects of some nanoparticles [61]. It has been shown that fullerene (C60) has immunosuppressive effects. C60 molecules are made up exclusively of carbon and are commonly used in nanotechnology for electronics, paints, and polymer composites. When they are incubated with mast cells, they reduce IgE-mediated signaling, ROS production, and degranulation. In the anaphylaxis mouse model, C60 prevents the secretion of histamine and prevents a decrease in body temperature, which usually occurs in mice after an allergy challenge. When C60 molecules are made like cylinders, they are called carbon nanotubes (CNTs) and are typically about 10 nanometers in diameter and several micrometers in length. This structure can be formed as single-walled or multi-walled pipes. Furthermore, it has been revealed that they have immunosuppressive effects. DCs exposed to lipopolysaccharides (LPS) and single-walled CNTs were less able to promote proliferation of T cells than DCs that were exposed to LPS alone. The mechanisms for the effect of single-walled CNTs on DC performance are not fully elucidated [62].

## 9. Conclusions

Nanotechnology is currently used for preventive and therapeutic applications. In the future, the use of nanoparticles with unique immunological properties will enable researchers to customize immune responses in new and unexpected ways. The size, shape, porosity, and hydrophobicity of the nanoparticles are important. Activation of CTL cells by nanoparticles can target tumors or virus-infected cells. Nanoparticles can be surrounded by viral antigens to enhance CTL activity. They also produce cytokines such as GM-CSF, IL-12, and IL-15. We can also use “suppressive nanoparticles” to control autoimmune diseases and prevent disease progression. In addition to the therapeutic applications of nanotechnology, nanosensors can help us to detect immune system diseases and discover new drugs. These emerging technologies offer new ways to differentiate immune cells and balance T-helper and T-Reg cells. These methods may also provide more effective medications in the future to regulate the immune system and reduce side effects. Briefly, nanotechnology will continue to provide insights into the nature of the immune response. The use of nanotechnology in immunology may also influence new strategies for the prevention or treatment of human diseases.

## Figures and Tables

**Table 1:** The size distribution of some immunological structures.

Immunological Structure	Size (nm)
Complement	1–5
Toll-like receptor (TLR)	2–10
T cell receptor (TCR)	2–10
The cluster of differentiation (CD) markers	2–10
Antibody	10–15
T cells	7000–12,000
B cells	7000–12,000
Neutrophils	10,000–12,000
Dendritic cells	10,000–22,000
Macrophages	10,000–22,000

**Table 2:** The relationship between nanoparticle size and bioactivity.

Nanoparticles	Size	Bioactivity
Dendrimer	<5 nm	Partition like small molecules and filter through the kidney
Polymer	10–20 nm	Escape the vasculature, infiltrate the tissues and lymphatics like proteins
DNA polyplex	50–100 nm	Penetrate the mucosal membranes and the skin and are taken up into cells
Liposome	>100 nm	Taken up mainly into phagocytic cells

**Table 3:** Some examples of nano-based vaccines.

Nano-Based Vaccines	Size Range	Mechanisms
Virus-like particles	15–30 nm	Repetitive antigen display, structural or molecular mimicry of the virus, particle size-dependent tissue penetration and trafficking to lymphatics, and TLR activation
MF59 ^a^	150–200 nm	Neutrophil, monocyte, and DC recruitment, antigen uptake, and the induction of humoral and TH1-type immune responses
W805EC ^b^	200–400 nm	Antigen uptake by and activation of epithelial cells and DCs, TLR2 and TLR4 activation, local cytokine production, mucosal antibody responses, and TH1, TH2, and TH17 cell responses
PLGA ^c^	100–200 nm	Encapsulation for sustained local antigens and co-mediator release
Nanogel	30–40 nm	Antigen entrapment in a hydrated nanogel matrix for slow release, delivery to APCs, and induction of tumor-specific T cells and antibody responses
Cationic liposomes	200–1000 nm	Encapsulation and targeted antigen delivery or uptake by APCs, and recruitment of monocytes to the injection site

^a^: MF59 is an immunologic adjuvant that uses a derivative of shark liver oil called squalene, ^b^: W805EC is an oil-in-water nano-emulsion-based adjuvant for an intranasally delivered vaccine, ^c^: poly(lactic-co-glycolic acid).

**Table 4:** The immunosuppressive effects of some nanoparticles.

Nanoparticles	Size Range	Mechanisms	Medical Application	Current Use
Fullerenes	0.5–1 nm	Suppression of mast cell and basophil degranulation	Allergy	In mice and in vitro
SWCNT ^a^	1–4 nm diameter; 1000–3000 nm length	Suppression of DC function	Inhalation exposure	In mice
MWCNT ^b^	10–20 nm diameter; 5000–15000 nm length	Suppression of T cell proliferation and function	Inhalation exposure	In mice

^a^: single-wall carbon nanotube, ^b^: multi-wall carbon nanotube.
